# Nicotinamide Mononucleotide Alleviates LPS-Induced Inflammation and Oxidative Stress *via* Decreasing COX-2 Expression in Macrophages

**DOI:** 10.3389/fmolb.2021.702107

**Published:** 2021-07-06

**Authors:** Jing Liu, Zhaoyun Zong, Wenhao Zhang, Yuling Chen, Xueying Wang, Jie Shen, Changmei Yang, Xiaohui Liu, Haiteng Deng

**Affiliations:** ^1^ MOE Key Laboratory of Bioinformatics, Center for Synthetic and Systematic Biology, School of Life Sciences, Tsinghua University, Beijing, China; ^2^ Shenzhen Hope Life Biotechnology Co., LTD, Shenzhen, China

**Keywords:** inflammation, macrophage, nicotinamide mononucleotide, NAD, COX-2, prostaglandin E_2_

## Abstract

Macrophage activation is an important process in controlling infection, but persistent macrophage activation leads to chronic inflammation and diseases, such as tumor progression, insulin resistance and atherosclerosis. Characterizing metabolic signatures of macrophage activation is important for developing new approaches for macrophage inactivation. Herein, we performed metabolomic analysis on lipopolysaccharide (LPS)-activated macrophages and identified the associated changes in metabolites. Notably, the cellular Nicotinamide adenine dinucleotide^+^ levels were decreased while NADPH was increased, proposing that NAD^+^ restoration can inhibit macrophage activation. Indeed, supplementation of nicotinamide mononucleotide (NMN) increased cellular NAD^+^ levels and decreased cytokine productions in LPS-activated cells. Quantitative proteomics identified that nicotinamide mononucleotide downregulated the expressions of LPS-responsive proteins, in which cyclooxygenase-2 (COX-2) expression was significantly decreased in NMN-treated cells. Consequently, the cellular levels of prostaglandin E_2_ (PGE_2_) was also decreased, indicating that NMN inactivated macrophages via COX-2-PGE_2_ pathway, which was validated in activated THP-1 cells and mouse peritoneal macrophages. In conclusion, the present study identified the metabolic characteristics of activated macrophages and revealed that NMN replenishment is an efficient approach for controlling macrophage activation.

## Introduction

Inflammation is an adaptive process when the human body constantly exposes to harmful stimuli (Medzhitov, 2008). Recent studies have shown that inflammation is intimately involved in tumor proliferation and migration as well as many other diseases (Balkwill and Mantovani, 2001; Homey et al., 2002). Macrophages play an important role in the initiation, maintenance, and resolution of inflammation through production of various cytokines and growth factors (Kasahara and Matsushima, 2001). Macrophage can be activated into M1 macrophages by IFN-γ or lipopolysaccharide, or M2 macrophage by IL-4 and IL-13 (Martinez and Gordon, 2014). Activated M1 macrophages promotes inflammation by secreting cytokines, such as interleukin-1 beta (IL-1β), interleukin-6 (IL-6), tumor necrosis factor-alpha (TNF-α), nitric oxide (NO) and prostaglandins (PGE_2_) (Fujiwara and Kobayashi, 2005). PGE_2_ that mediates inflammatory response is synthesized from arachidonic acid by cyclooxygenases (COX-1 and COX-2) (Ricciotti and FitzGerald, 2011). COX-1 and COX-2 are the targets of anti-inflammation, and are inhibited by nonsteroidal anti-inflammatory drugs (NSAIDs) (Kerola et al., 2009; Tóth et al., 2013). The prolonged administration of NSAIDs causes many undesirable side effects such as gastrointestinal bleeding (Laine, 2003). Finding new anti-inflammatory molecules is important for inflammation management.

Nicotinamide adenine dinucleotide (NAD) is one of the most important molecules in cells by participating in hundreds of redox reactions as a coenzyme and by mediating multiple signaling pathways (Chiarugi et al., 2012; Katsyuba and Auwerx, 2017). Numerous studies demonstrate the therapeutic benefits of NAD^+^ precursor supplementation to aging-associated disorders in the nervous system, liver and kidney diseases and cardiac diseases (Pillai et al., 2005; Lingor et al., 2012; Cantó et al., 2015; Morigi et al., 2015; Zong et al., 2021). NAD^+^ is generated from the *de novo* pathway from tryptophan, the Preiss-Handler pathway from nicotinic acid (NA), and/or the salvage pathway from nicotinamide (NAM), nicotinamide riboside (NR) or nicotinamide mononucleotide (NMN) (Bieganowski and Brenner, 2004; Yang and Sauve, 2016). Recently, it has been reported that the excessive oxidative stress during aging and chronic inflammation led to NAD^+^ depletion (Schultz and Sinclair, 2016; Covarrubias et al., 2020). It has been amply documented that the administration of NMN increases the cellular NAD^+^ and alleviated many diseases symptoms (Yoshino et al., 2011; Yamamoto et al., 2014; Guan et al., 2017). In addition, adipose tissue inflammation can be suppressed by the long-term administration of NMN (Mills et al., 2016). Hence, we aim to determine whether NMN supplementation can alleviate inflammation through increasing cellular NAD^+^ level.

In the present work, we performed metabolomic analysis on LPS-activated macrophages and identified the associated metabolic signatures. We found that NMN supplementation efficiently alleviated LPS-induced inflammation via decreasing secretion of pro-inflammatory cytokines. Proteomics analysis showed that inflammation-related pathways were suppressed and COX-2 expression was downregulated in NMN-treated cells. These results confirm that NAD^+^ precursors nicotinamide mononucleotide (NMN) suppress inflammation, suggesting that NMN is an effective treatment for chronic inflammation.

## Materials and Methods

### Cell Culture and Drug Treatment

The mouse macrophage cell line RAW264.7 was a generous gift from Xin Lin Laboratory, School of Life Sciences, Tsinghua University, Beijing, China. Human monocyte cell line THP-1 and human kidney cell line 293T were obtained from the Cell Bank of Chinese Academy of Science (Shanghai, China). Briefly, peritoneal macrophages, RAW264.7 and 293T cells were cultured in Dulbecco Modified Eagle Medium (DMEM) medium (Wisent, Montreal, QC) with 10% fetal bovine serum (FBS) (Wisent, Montreal, QC) and 1% penicillin/streptomycin (Wisent, Montreal, QC), respectively. And THP-1 cells were cultured in RPMI 1640 Medium (Wisent, Montreal, QC) with 10% FBS and 1% penicillin/streptomycin.

For macrophage activation, THP-1 cells were treated with 10 ng/ml PMA (Beyotime, S1819) for 12 h to obtain PMA- activated THP-1 macrophages. Then PMA-activated THP-1, RAW264.7 and Peritoneal Macrophages were seeded in multiwell plates. After seeding for 24 h, cells were treated with nicotinamide mononucleotide (NMN, 500 μM) for 12 h or 24 h in the absence or presence of lipopolysaccharides (LPS, 100 ng/ml) in the culture medium.

### Metabolomic Analysis

The analysis was performed following a protocol as previously reported (Tang et al., 2016). Briefly, the cells were covered with pre-chilled 80% methanol after washed by PBS and plated in a refrigerator for 1 h at –80 C. Then, the cells were scraped in 80% methanol on dry ice and centrifuged at 12,000 rpm for 20 min at 4°C. The supernatant was dried by speedvac and dissolved in 80% methanol and analyzed by LC–MS/MS. The TSQ Quantiva™ Triple Quadrupole Mass Spectrometer with Ultimate 3000 (Thermo Fisher Scientific, Waltham, MA, United States) was employed for targeted quantitative analysis in positive and negative ion switching mode. For untargeted metabolites profiling, the Q-Exactive Mass Spectrometer (Thermo Fisher Scientific, Waltham, MA, United States) was employed. Retention time on the chromatograms and m/z determined by TraceFinder software (version 3.2, Thermo-Fisher Scientific) were applied to identify metabolites.

### Macrophage Preparation

Peritoneal macrophages were isolated from C57BL/6 J mice (6–8 weeks) after 1 ml intraperitoneal starch induction for 3 days. The mice were sacrificed and the peritoneal cavity was flushed with 10 ml ice-cold PBS. And the peritoneal cells were collected by centrifugation at 800 rpm for 5 min and seeded on a Petri dish in a 37°C humidified incubator containing 5% CO_2_. After 2 h, the plates were gently washed five times with warm PBS to remove the non-adhesive cells. The adherent cells were used as the peritoneal macrophages in the present experiments.

### Quantitative RT-PCR Assay

The total RNA was isolated from cell lysate using TRIzol extraction (Tiangen, Beijing, China). Then, cDNA was synthesized from 2 μg total RNA using the reverse transcription system (CWBIO, Beijing, China) following the manufacturer’s manual. Q-PCR was performed with the Roche LightCycler 96 System (Roche, Basel, Switzerland) with SYBR green (CWBIO, Beijing, China) and ACTB was used as an internal control. Primer sequences in this study were listed in Supplementary Table 1.

### Enzyme Linked Immunosorbent Assay

To collect peritoneal lavage fluid, the mouse peritoneal cavity was flushed with 10 ml ice-cold PBS and immediately kept on ice. Lavage fluid was centrifuged at 800 rpm for 5 min and the supernatant was collected for analysis. Similarly, cell culture supernatants of RAW264.7 and THP-1 were also collected. ELISA for IL-6 (Dakewe Biotech Co., Ltd) and IL-1β (ThermoFisher# 88-7013-22) was respectively performed using two different kits following the manufacturer’s manuals.

### Sample Preparation for Proteomics Analysis

Cells were scraped from the 10 cm dishes and resuspended in 400 µl 8M urea in phosphate buffer saline (PBS) (Wisent, Nanjing, China), 1× protease inhibitor cocktail. The lysate was placed on ice for 30 min and centrifuged at 12,000 rpm for 20 min to collect the supernatant. The protein concentration was determined by using a BCA protein assay kit (Solarbio, Beijing, China). Each sample (100 ug protein) was reduced with 5 mM dithiothreitol (DTT) at room temperature for 1 h and then alkylated with 12.5 mM iodoacetamide (IAM) at room temperature. After dilution with 100 mM TEAB in a 1:5 ratio, proteins were digested with sequencing-grade-trypsin (Promega, Madison, WI, United States) overnight at 37 C. The peptides were desalted using Sep-Pak desalting columns (Waters, Milford, MA, United States). Eluted peptides were dried in SpeedVac and labeled with tandem mass tags (TMT) 6-plex reagents (Thermo Fisher Scientific, Waltham, MA, United States) overnight at 4 C. The TMT6 labeled peptides from different samples were mixed together after terminated by adding 4 µl hydroxylamine (5%) and desalted by Sep-Pak C18 Vac cartridges. The peptides were separated with a UPLC 3000 system (Thermo-Fisher Scientific) with an XBridge C18 RP column (Waters, Milford, MA), and eluted by gradient buffer. The sample were divided into 48 fractions to dry by speedvac and recombined into 12 fractions. The peptides were extracted twice with 0.1% formic acid and analyzed by LC-MS/MS.

Quantitative proteomics was performed on an Orbitrap Fusion LUMOS Tribrid mass spectrometer (Thermo Fisher Scientific, Waltham, MA, United States). Data from MS/MS spectra of each LC-MS/MS run were searched using the Sequest HT in Proteome Discoverer (PD) 2.1 software (Thermo Fisher Scientific, Waltham, MA, United States).

### Western Blot Analysis

Cells were lysed in RIPA buffer (Solarbio, Beijing, China), supplemented with 1% Protease Inhibitor Cocktail (Selleck, Shanghai, China) and 1mM PMSF. The lysate was plated on ice for 30 min and centrifuged at 12,000 rpm for 20 min. The protein concentration was determined by using a BCA protein assay kit. Equal amounts of protein were separated using a 10% SDS–PAGE gel and transferred onto a polyvinylidene difluoride (PVDF) membranes (Millipore, Billerica, MA, United States). The membranes were blocked in 5% milk in TBST buffer (20 mmol/L Tris-HCl, 150 mmol/L NaCl, and 0.1% Tween 20) for 1 h at room temperature. Then, the blocked membranes were incubated with primary antibody (COX-2, Cell Signaling Technology, 1:2000, 12282S) and HRP-conjugated secondary antibodies (1:2000 dilution, Cell Signaling Technology). Finally, quantification of protein bands were measured by densitometry using Bio-Red software.

### Establishment of Stable COX-2-Overexpression and COX-2-Knockdown Cell Lines

To construct stable COX-2-Overexpression cell lines, mouse *Cox2* cDNA was obtained from Ptgs2 (NM_011198) Mouse Tagged ORF Clone (Clone ID MR227684, OriGene). The cDNA was cloned into the plasmid pLVX-IRES-ZsGreen1 to create the pLVX-COX-2-IRES-ZsGreen1 vector. Empty pLVX-IRES-ZsGreen1 was used as negative control. Recombinant construct with three helper plasmid pLP1, pLP2 and VSVG were transiently transfected into 293T cells using Lipofectamine™ 3000 (Invitrogen, Carlsbad, CA, United States) following the manufacturer’s instructions. After 48 h, the supernatant was collected and lentivirus particles were concentrated with 4 M NaCl and PEG6000. Then fresh virus was used to infect RAW264.7 cells with 10 ng/µl polybrene. GFP positive polyclone cells were sorted by a flow cytometer to generate stable cell lines. The polyclone cells were measured by western blotting.

To establish stable COX-2-Knockdown cell lines, two shRNA sequences were chosen for targeting COX-2 by NCBI BLAST (Tiedemann et al., 2012). The double-stranded oligonucleotides were annealed and inserted into plasmid pLL3.7 lentivirus vectors. The transfection and cell line sorting procedures were same as the ones used for construction of overexpression cell lines. Primer sequences in this study were listed in Supplementary Table 1.

### Detection of Cellular Prostaglandin E_2_ Levels

For PGE_2_ extraction, cells were treated with NMN or LPS for 24 h and then washed by ice-cold PBS. Cells were scraped with 15% precooled-methanol on dry ice. And the supernatant was collected for enrichment using a SPE column (Waters, Milford, MA) following the manufacturer’s manual. The eluted sample was dried by Nitrogen gun and redissolved in 30% acetonitrile before LC-MS/MS analysis. Each group was repeated four times to ensure accuracy.

### Statistical Analysis

GraphPad Prism (version 8.0) software and R (version 3.5.2) were used for statistical analysis. All experiments were repeated at least three times. Student’s *t*-test was used for comparisons of only two group and one-way ANOVA was used to compare three or more groups. *P* values of <0.05 were considered significant (*p < 0.05; **p < 0.01; ***p < 0.001).

## Results

### Metabolomic Analysis Revealed That Cellular NAD^+^ Level Was Decreased in LPS-Activated Macrophages

**FIGURE 1:**
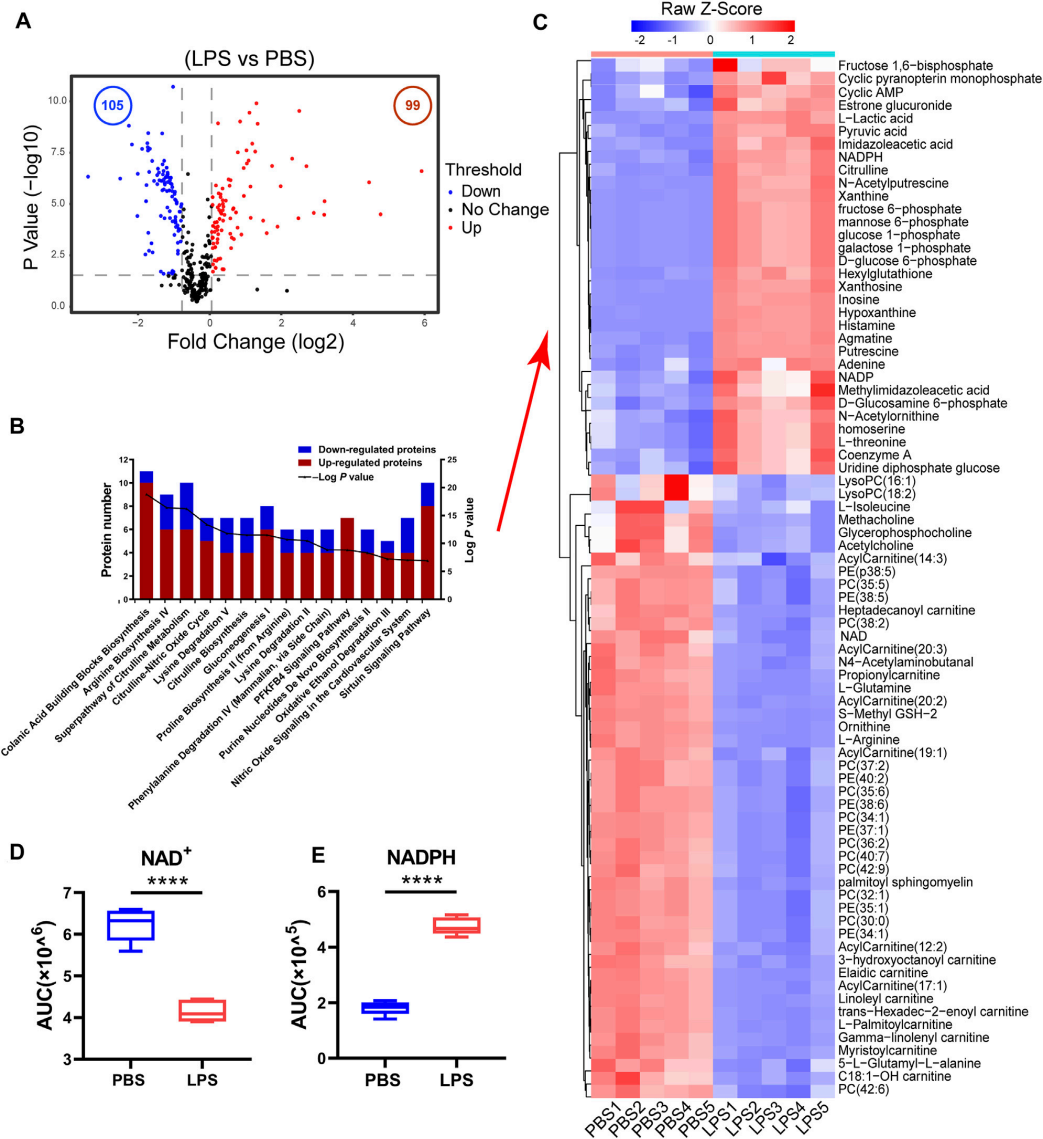
The cellular NAD^+^ content decreased in LPS-activated macrophages. **(A)** A volcano plot of metabolites in LPS-treated cells compared with control cells. Blue and red dots represent the decreased and increased metabolites (*p*-values < 0.05, ratios ≤0.75 or ≥1.33), respectively. **(B)** Functional enrichment analysis of the changed metabolites. The red bar represented the number of increased metabolites while the blue represented the number of decreased ones in different biological processes (LPS-treated cells versus untreated cells > 1.33-fold or < 0.75, *p* < 0.05). **(C)** Heatmap clustering analysis of the changed metabolites selected based on IPA analysis in LPS-treated cell as compared to those in untreated cells. **(D,E)** NAD^+^ and NADPH levels from the metabolomics data (****p < 0.0001, **p < 0.01, *p < 0.05, *n* = 5, mean ± SEM).

To understand the metabolic changes of macrophages in response to LPS treatment, we performed metabolomic analysis in five biological replicates in RAW264.7 cells. We identified 458 ionic species putatively annotated by mapping their measured masses to mouse metabolites (Supplementary Table 2). Metabolomics analysis showed that the levels of 99 metabolites (fold change >1.33 and *p* < 0.05) were increased while the levels of 105 metabolites (fold change <0.75 and *p* < 0.05) were decreased in LPS-treated cells in comparison with control group. The data were presented in a volcano plot (Figure 1A, Supplementary Table 3). Heatmap analysis was performed on normalized data to identify the changed metabolites in LPS-treated cells as compared with control group (Supplementary Figures 1A,B).

The ingenuity pathway analysis of the significantly changed metabolites demonstrated that Colanic Acid Building Blocks Biosynthesis and Arginine Biosynthesis IV were the most activated (*p*-values < 0.05) canonical pathways in LPS-treated cells (Supplementary Table 4). Other activated pathways included Superpathway of Citrulline Metabolism, Citrulline-Nitric Oxide Cycle, Lysine Degradation V and Citrulline Biosynthesis (Figure 1B). Based on IPA analysis, 81 metabolites were displayed in a heatmap (Figure 1C, Supplementary Table 5), whose levels were either increased or decreased in the LPS-treated cells, designated as LPS-responsive metabolites. Fructose 1,6-bisphosphate, galactose-1-phosphate, glucose-1-phosphate, GTP, mannose-6-phosphate, NADP and NADPH were enriched in Colanic Acid Building Blocks Biosynthesis pathway (Figure 1C). And we found the levels of citrulline and oxoglutaric acid associated with Citrulline-Nitric Oxide Cycle were also increased in LPS-treated RAW264.7 cells whereas levels of 3-hydroxyoctanoyl carnitine, Linoleyl carnitine, Heptadecanoyl carnitine and Propionylcarnitine related to carnitine metabolism were decreased. Noticeably, the cellular NAD^+^ level was significantly decreased in LPS-activated macrophages, while NADPH was increased (Figures 1D,E). NADPH is an important factor in macrophage activation for RNS and ROS production. Consequently, the increase of NADPH may correlate to a decrease in cellular NAD^+^ level. In addition, we found that the content of NADH and NADP was decreased after LPS stimulation in RAW264.7 cells (Supplementary Figures 1C,D). Taken together, these results suggest that NAD^+^ restoration can inhibit LPS-induced macrophage activation.

### Nicotinamide Mononucleotide Supplementation Increased NAD^+^ Levels and Inhibited LPS-Induced Inflammation in Macrophages

**FIGURE 2:**
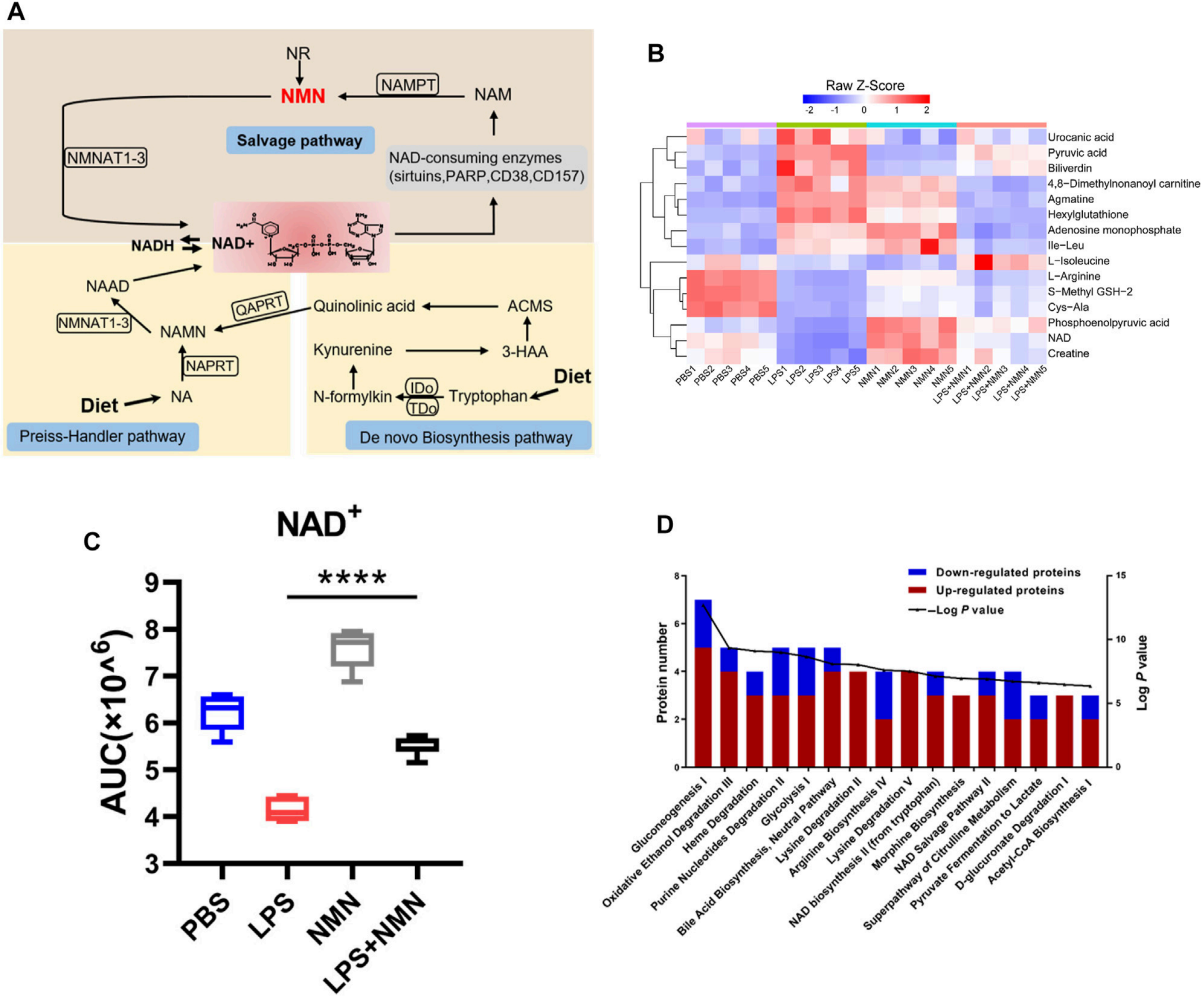
NMN supplementation increased cellular NAD^+^ levels. **(A)** A schematic view of NAD^+^ biosynthesis. **(B)** Heatmap clustering analysis of 15 metabolites whose expressions were antagonized by NMN in LPS treated- macrophages (>1.33-fold or <0.75-fold; *p*-value< 0.05). **(C)** Changes in NAD^+^ levels in LPS-, NMN-, LPS/NMN co-treated groups from the metabolomics data (****p < 0.0001, **p < 0.01, *p < 0.05, *n* = 5, mean ± SEM). **(D)** Ingenuity pathway analysis. The red bar represented the number of increased metabolites, and while the blue represented the number of decreased ones in different biological processes. (LPS/NMN co-treated group versus LPS treated group >1.33 or <0.75-fold, *P* < 0.05).

The three pathways for NAD^+^ synthesis were *De novo* synthesis pathway from tryptophan, the preiss-handler pathway from NA and the salvage pathway from NAD^+^ precursors NAM, NR and NMN as shown in Figure 2A. As shown in Supplementary Figures 2A, NMN treatment antagonized the changes in metabolites in LPS-treated RAW264.7 cells. Out of 81 LPS-responsive metabolites, 15 were reversed in NMN treated- macrophages (Figure 2B, Supplementary Table 6). Consistently, we found that NMN supplementation restored the NAD^+^ levels in LPS-treated macrophage (Figures 2B,C). IPA analysis revealed that metabolites associated with Gluconeogenesis I, Oxidative Ethanol Degradation III and Heme Degradation were highly enriched. Levels of L-Isoleucine, S-Methyl GSH-2, NAD and creatine were all increased in LPS- and NMN-cotreated cells as compared with LPS-treated cells, whereas levels of 4,8-Dimethylnonanoyl carnitine, Adenosine monophosphate and Pyruvic acid were decreased (Figure 2D). To further explore the relationship between NMN treatment with the NAD^+^ levels in LPS-stimulated macrophages, the effect of different concentrations of NMN was examined. As Supplementary Figure 2B showed that NAD^+^ content increased with increasing NMN concentration in LPS-treated RAW264.7 cells. These results suggested that NMN suppressed LPS-induced macrophage activation by restoring the levels of NAD^+^.

**FIGURE 3:**
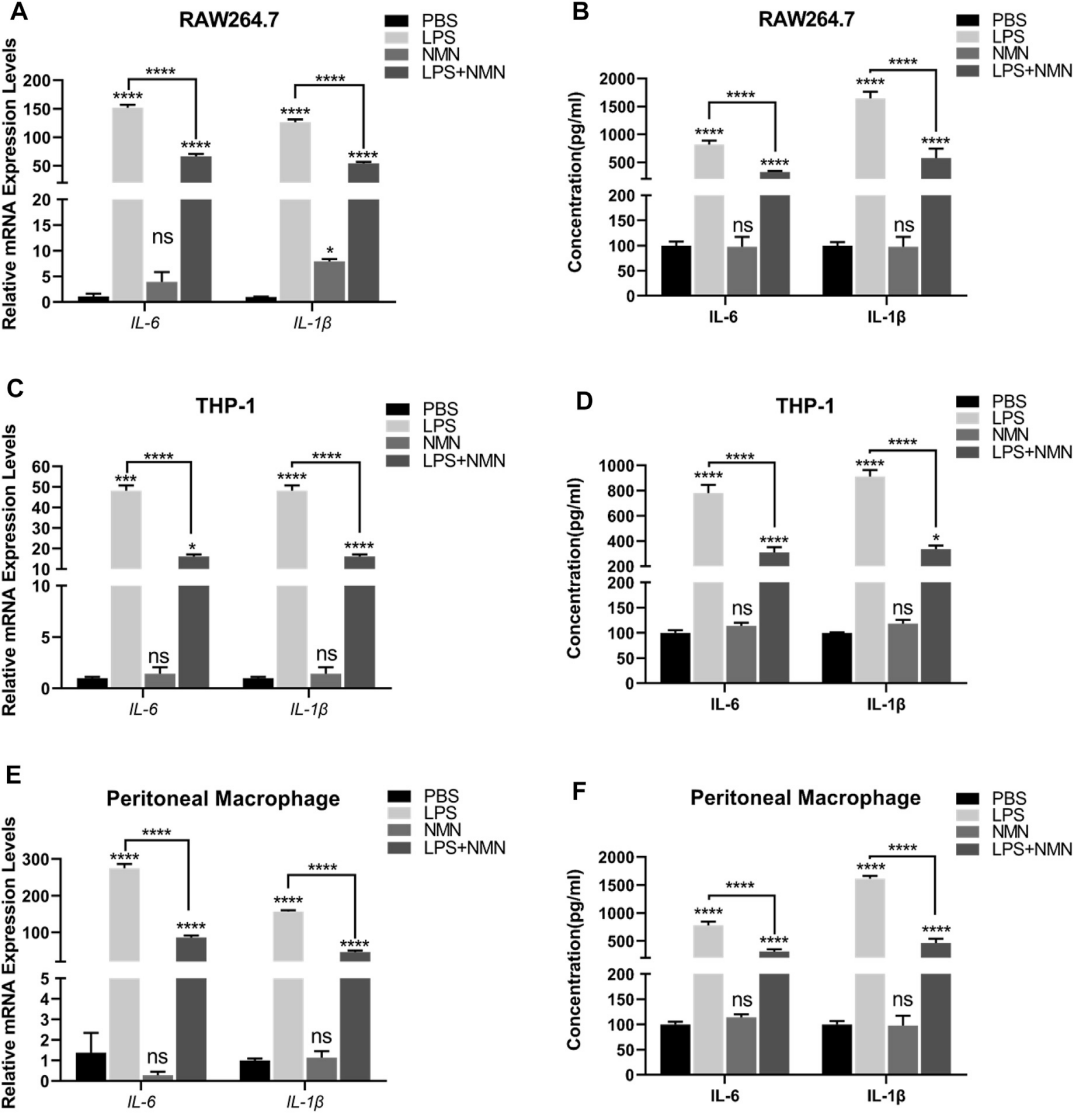
NMN suppressed inflammatory cytokines in LPS-induced macrophages **(A,C,E)** The mRNA expressions of *IL-6* and *IL-1β* were examined by RT-qPCR in untreated, LPS-treated, NMN-treated, LPS/NMN co-treated RAW264.7 cells, THP-1 cells and mouse peritoneal macrophages. **(B,D,F)** The ELISA results of IL-6 and IL-1β in untreated, LPS (100 ng/ml)-treated, NMN (500 µM)-treated and LPS-NMN-treated RAW264.7 cells, THP-1 cells and mouse peritoneal macrophages. The experiment was performed in triplicates and repeated three times. *P* values were calculated using one way ANOVA test. ****p < 0.0001, **p < 0.01, *p < 0.05; mean ± SEM.

To further investigate whether NMN inhibits LPS-induced inflammation, we established a cellular model using LPS-treated RAW264.7 cells. RAW264.7 cells were treated with LPS/NMN for 12 h, respectively. Next, we collected the cells and supernatants for Q-PCR and ELISA analysis. IL-6 and IL-1β are pro-inflammatory cytokines as an index of inflammation (Curran et al., 2005; Shapouri-Moghaddam et al., 2018). Therefore, we examined whether LPS-induced inflammation was attenuated by NMN supplementation by detecting IL-6 and IL-1β content, showing that mRNA expressions of *IL-6* and *IL-1β* were all decreased in LPS- and NMN-cotreated RAW264.7 cells (Figure 3A). Consistent with the intracellular changes of *IL-6* and *IL-1β* mRNA, extracellular secretion of IL-6 and IL-1β by RAW264.7 cells was markedly decreased by NMN supplementation (Figure 3B). To further explore the function of NMN in suppressing inflammation, we used different concentrations of NMN to treat RAW264.7 cells. The mRNA expression of *IL-6* and *IL-1β* gradually decreased in LPS- and NMN-cotreated RAW264.7 cells as the NMN concentration increased (Supplementary Figures 3A,B). To validate that NMN suppresses LPS-induced inflammation, we performed the similar experiments in THP-1 cells and mouse peritoneal macrophages. Similarly, NMN treatment decreased the mRNA expression of *IL-6* and *IL-1β* and extracellular secretion of IL-6 and IL-1β in both cells (Figures 3C–F). Thus, these data indicate that NMN alleviates LPS-induced inflammation in macrophages and effectively inhibits secretion of IL-6 and IL-1β.

### Proteomic Analysis Showed That NMN Decreases the Expression of Inflammation-Related Proteins and Inhibits the Inflammation-Related Pathway

To investigate the effects of NMN treatment on LPS-activated macrophages, we performed proteomic analysis using Tandem Mass Tag (TMT)-based quantitation. We identified 8,464 proteins, of which 6,881 proteins were confidently identified (false discovery rate of protein <0.01, score ≥ 5, unique peptide ≥ 2) by the Protein Discovery 2.1 (Supplementary Table 7).

**FIGURE 4:**
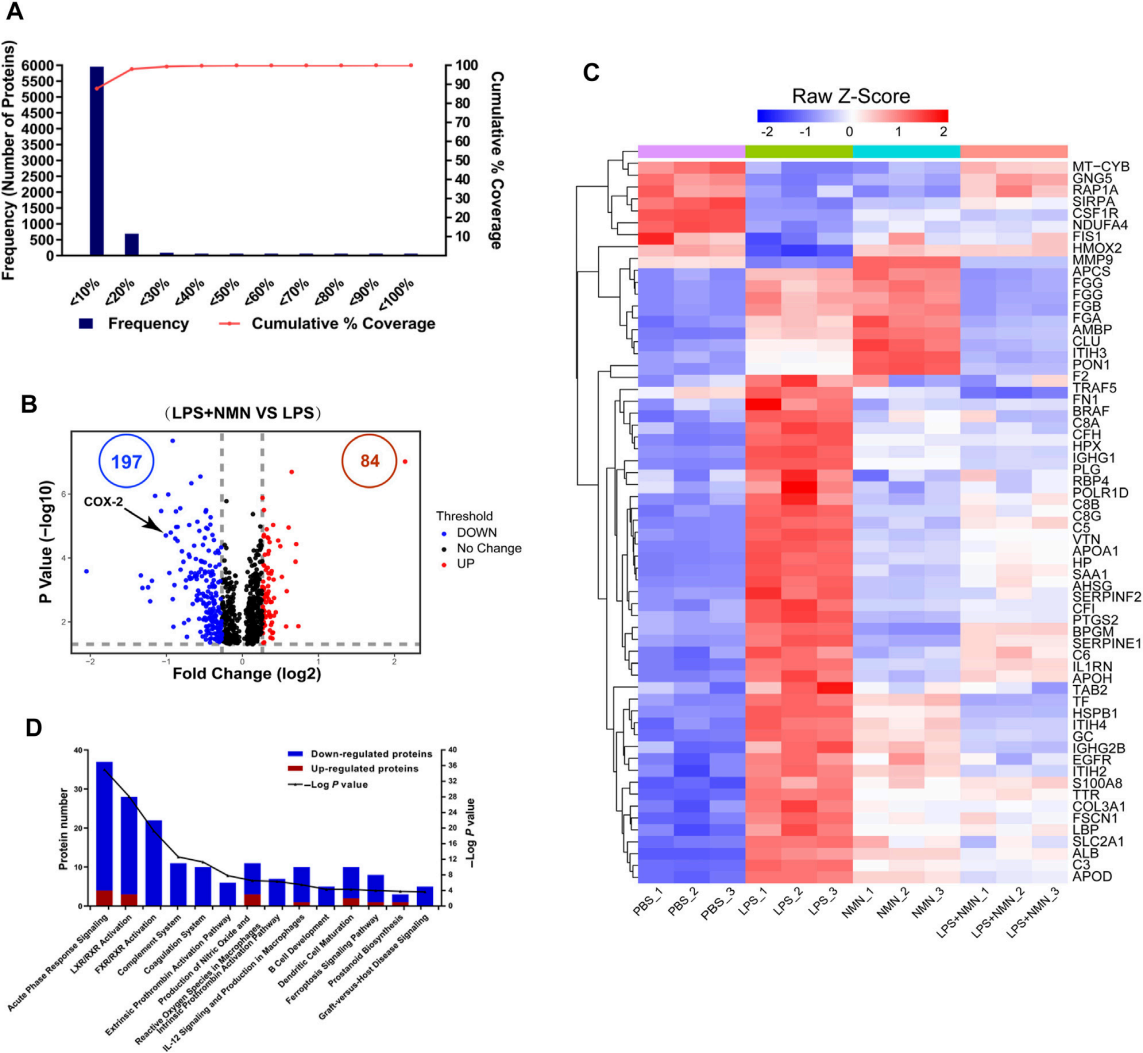
Functional classification of differentially expressed proteins (DEPs) from proteomics analysis of LPS-NMN-treated RAW264.7 cells as compared with LPS-treated cells. **(A)** Experimental variations of proteomics analysis in this study. **(B)** The volcano plot shows differentially expressed proteins (DEPs) in LPS-NMN-treated cells as compared with LPS-treated cells. The red and blue dots indicate the significantly changed proteins with fold-change > 1.2 or <0.83 and *p* value <0.05. COX-2 is highlighted. **(C)** 61 differentially expressed proteins were found based on the filter conditions that fold change (LPS and NMN co-treated cells versus LPS-treated cells) > 1.2 or <0.83-fold in LPS-induced activated macrophages. **(D)** The ingenuity pathway analysis for DEPs in LPS-NMN-treated cells as comparing with LPS-treated cells. The red bar represented up-regulated proteins, and blue represented down-regulated (LPS-treated cells versus untreated cells > 1.2-fold or < 0.83, *P* < 0.05).

To understand the changes in protein expression associated with LPS-treated RAW264.7 cells, we first compared protein expressions between the LPS-treated group with untreated group. Population statistics analysis was applied to determine the threshold cut-off values for differentially expressed proteins. According to reports in the literature (Suriyanarayanan et al., 2018), percentage variations corresponding to 88% coverage were viewed as threshold cut-off (Figure 4A). The criteria of upregulated proteins were ratios ≥ 1.2 and p-values <0.05 and those for downregulated proteins were ratios ≤ 0.83 and *p*-values <0.05, respectively. Among differentially expressed proteins, 728 proteins were upregulated while 510 proteins were downregulated in LPS-treated cells compared with untreated group (Supplementary Figure 4A, Supplementary Table 8). Based on these differentially expressed proteins (DEPs), we further conducted IPA analysis (Supplementary Figure 4B). Pathways with more than 100 differentially expressed proteins were shown in a heatmap (Supplementary Figure 4C), showing that the proteins related to LXR/RXR activation, acute phage response signaling and IL-12 signaling and production in macrophage were upregulated in LPS-treated cells, consisting with LPS induced macrophage activation.

Using the similar approach, we identified that 84 proteins were upregulated while 197 were downregulated in LPS/NMN co-treated cells as compared with LPS-treated cells (Figure 4B, Supplementary Table 9). We identified 61 differentially expressed proteins by adding NMN in activated macrophages (Figure 4C, Supplementary Table 10). We found that the proteins related to inflammatory response such as RELL1, PTGS2, FGA, FGB and igkv12-44 were all decreased in LPS/NMN co-treated cells as compared with LPS-treated cells, which suggested that NMN inhibited macrophage activation. Subsequently, we performed IPA analysis and the results showed that NMN decreased the protein expression related to the pathway of acute phase response signaling; LXR/RXR Activation, FXR/RXR Activation and Complement System, which were activated in LPS-activated macrophages (Figure 4D). NMN treatment also suppressed other inflammation-associated pathways such as prostanoid biosynthesis, LPS/IL-1 mediated inhibition of RXR function, IL-6 signaling and NF-κB signaling. These results indicate that NMN decreases the expression of inflammation-related proteins and inhibits the inflammation-related pathway.

### NMN Reduced LPS-Induced Inflammation *via* Decreasing COX-2 Expression to Inhibit Prostaglandin E_2_ Synthesis

**FIGURE 5:**
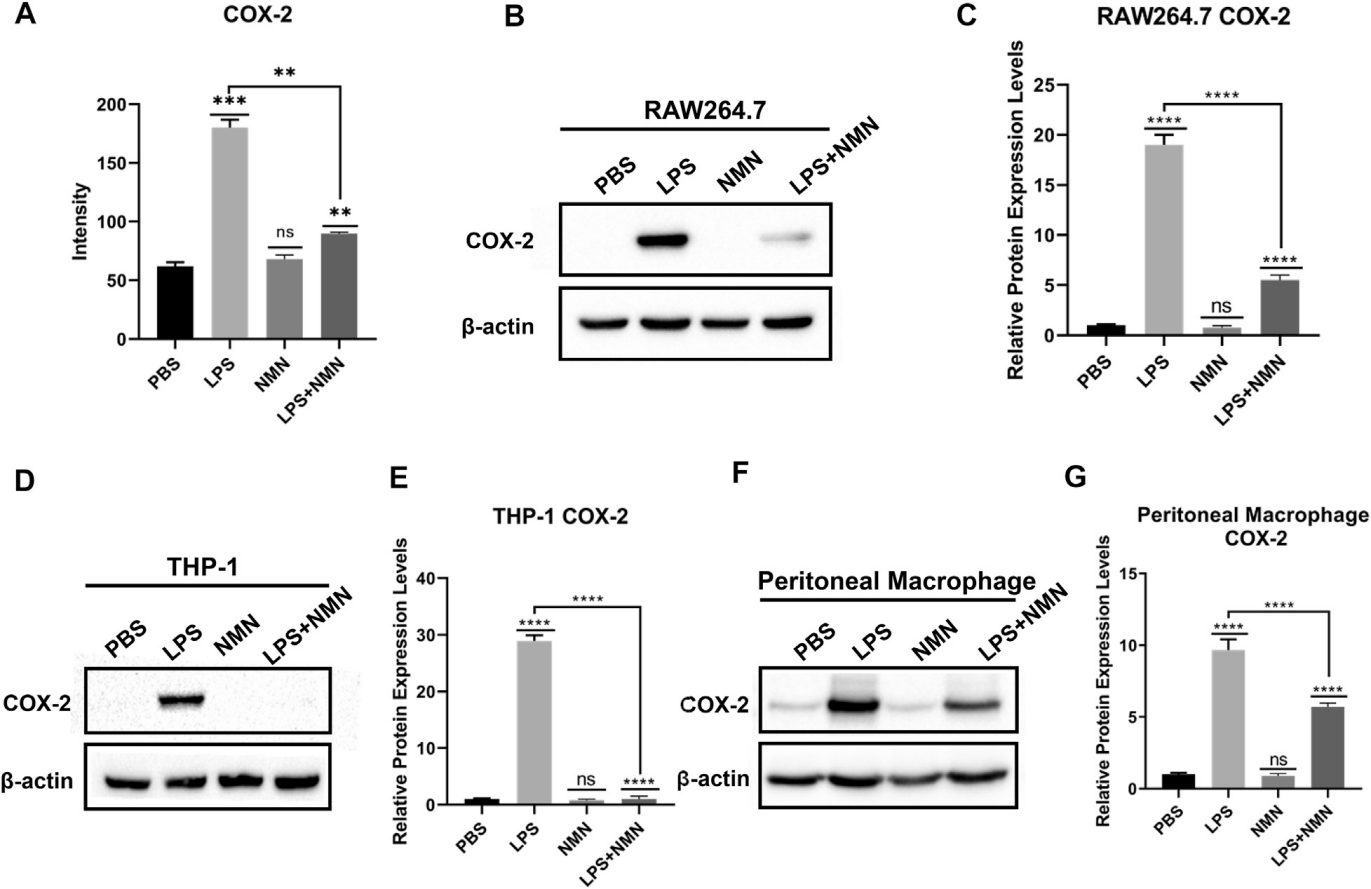
NMN decreased cellular COX-2 expression in RAW264.7 cells, THP-1 cells and mouse peritoneal macrophages. **(A)** COX-2 levels from the proteomics data. **(B,C)** COX-2 expression in RAW264.7 cells was analyzed by western blotting. **(D,E)** COX-2 expression in THP-1 cells was analyzed by western blotting. **(F,G)** COX-2 expression in peritoneal macrophages was analyzed by western-blotting. The experiment was performed in triplicates and repeated three times. *P* values were calculated using one way ANOVA test. ****p < 0.0001, **p < 0.01, *p < 0.05; mean ± SEM.

Among the downregulated proteins, COX-2 expression was decreased in LPS/NMN co-treated cells as compared with LPS-treated cells by proteomics analysis (Figures 4B, 5A). To confirm this finding, we performed western-blot analysis in RAW264.7 cells, THP-1 cells and mouse peritoneal macrophages, and indeed, COX-2 expression was remarkably reduced with NMN supplementation in LPS-treated cells (Figures 5B–G). Western-blot results also showed that with increasing concentrations of NMN, the expression of COX-2 gradually decreased in LPS-induced RAW264.7 cells (Supplementary Figure 5A). We further investigated whether NMN reduced the synthesis of COX-2 mRNA or promoted COX-2 degradation by Q-PCR in RAW264.7 cells. As the result showed in Supplementary Figure 5B, the mRNA level of COX-2 was reduced in LPS/NMN co-treated cells as compared with LPS-treated cells. We also treated RAW264.7 cells with cycloheximide to inhibit protein synthesis, showing that there were no changes in the presence or absence of NMN (Supplementary Figure 5C). Overall, these results indicate that NMN alleviates LPS-induced inflammation by decreasing COX-2.

**FIGURE 6:**
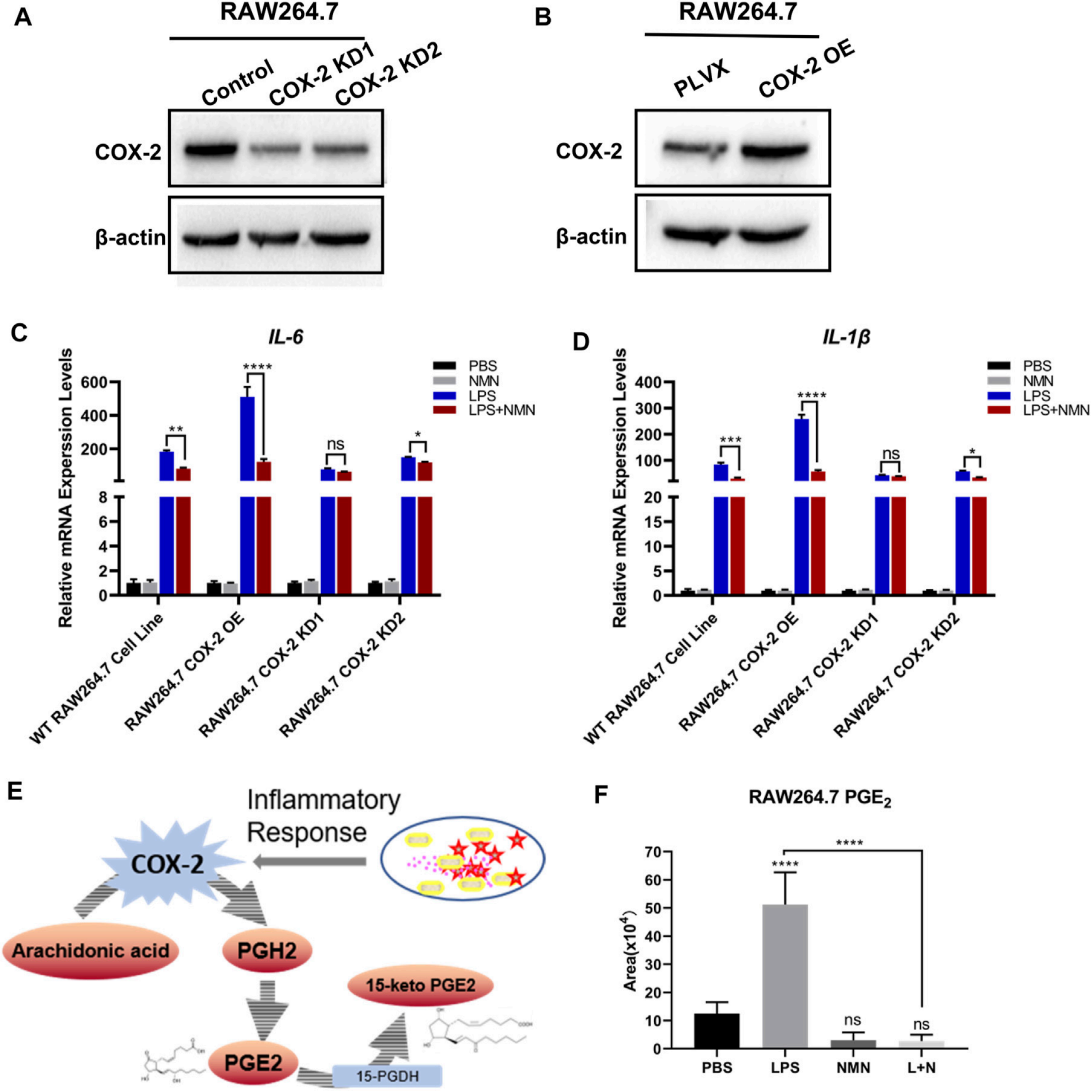
NMN alleviated LPS-induced inflammation *via* decreasing COX-2 expression and inhibiting prostaglandin E_2_ synthesis. **(A)** Western blotting of COX-2 revealed that the expression of COX-2 was reduced in RAW264.7-COX-2-KD cells. *β*-actin was used as a control. **(B)** Western blot analysis confirmed COX-2 overexpression in RAW264.7 cells. **(C,D)** The mRNA levels of *IL-6* and *IL-1β* were detected by RT-qPCR (*n* = 3) in WT RAW 264.7, RAW264.7-COX-2-OE, RAW264.7-COX-2-KD1 and RAW264.7-COX-2-KD2 cell lines under NMN or LPS stimulated, respectively. **(E)** Schematic diagram depicts the biosynthesis and metabolism of PGE_2_. **(F)** The levels of PGE_2_ in untreated, LPS (100 ng/ml)-treated and LPS-NMN (500 µM) co-treated RAW264.7 cells were analyzed by LC-MS/MS (*n* = 5). P values were calculated using one way ANOVA test; ****p < 0.0001, **p < 0.01, *p < 0.05; Data are shown as means ± SEM.

To further investigate whether NMN inhibit LPS-induced inflammation by reducing COX-2 expression, we treated RAW264.7 cells with the COX-2 inhibitor, celecoxib. As shown in Supplementary Figure 6A, the mRNA expression of *IL-6* and *IL-1β* had no change in LPS plus NMN co-treated cells as compared with LPS-induced cells in the presence of celecoxib, suggesting that COX-2 played a critical role in the process of NMN inhibit LPS-induced inflammation. Therefore, we established stable cell lines in which COX-2 was knocked down or overexpressed in RAW264.7 cells as confirmed by Q-PCR (Supplementary Figures 6B,C) and western blotting (Figures 6A,B). Upon LPS stimulation, the Q-PCR results showed that the mRNA expressions of *IL-6* and *IL-1β* were higher in COX-2-OE-cells than those in wild-type RAW264.7 cells (Figures 6C,D). This demonstrated that COX-2 promoted RAW264.7 inflammation responses. On the other hand, we revealed that NMN had no effects on the expressions of *IL-6* and *IL-1β* in LPS-induced COX-2- knockdown cells, whereas the inhibitory effects of NMN in *IL-6* and *IL-1β* expression was higher in COX-2-OE-cells after LPS treatment (Figures 6C,D). Additionally, the change law of contents of NAD^+^ and NADPH in COX-2-OE-cell and COX-2-KD-cell is very similar to wild-type RAW264.7 cells after LPS/NMN stimulation (Supplementary Figures 6D,E). Taken together, these results showed that NMN suppressed LPS-induced inflammation by decreasing COX-2 expression.

In cells, COX-2 converts arachidonic acid (AA) to prostaglandin endoperoxide H2 (PGH2). Then PGH_2_ is metabolized to PGE_2_ by COX-2.15-hydroxyprostaglandin dehydrogenase (15-PGDH) is the key enzyme in the biodegradation of PGE_2_. (Figure 6E). Our recent study has demonstrated that NAD^+^ precursors prevented ROS-mediated 15-PGDH degradation (Wang et al., 2018) and NMN decreases the level of PGE_2_ in TGF-β1-treated LX-2 cells to inhibit hepatic stellate cell (HSC) activation (Zong et al., 2021). In the present study, NMN supplementation has no effects on 15-PGDH expression. Therefore, we proposed that NMN decreased the level of PGE_2_ in LPS-activated RAW264.7 cells by downregulating COX-2 expression. We examined the level of PGE_2_ in LPS-treated, NMN-treated and LPS-NMN-cotreated cells by LC-MS/MS analysis. The results showed that the level of PGE_2_ was increased by LPS treatment, but when NMN was supplemented, it reduced the level of PGE_2_ (Figure 6F). When we treated RAW264.7 cells with different concentrations of NMN ranging from 100 μM to 1 mM, the level of PGE_2_ were also decreased to varying degrees stimulated by LPS (Supplementary Figure 6H). To further investigate the relationship between PGE_2_ with LPS-induced inflammation, we treated RAW264.7 cells with different concentrations of PGE_2_. As shown in Supplementary Figures 4E,F, the mRNA expression of *IL-6* and *IL-1β* was dependent on the concentrations of PGE_2_. These results show that NMN alleviates LPS-induced inflammation via decreasing COX-2 expression and inhibiting prostaglandin E_2_ synthesis.

## Discussion

Macrophages play an important role in inflammatory process and immune responses (Sica et al., 2008). The pro-inflammatory cytokines IL-6 and IL-1β secreted by macrophages are the measures of inflammatory response. In this study, we revealed the metabolic and proteomic signatures of macrophage activation and demonstrated that NMN supplementation efficiently alleviated LPS-induced inflammation. Multi-omics analysis revealed the global effects of LPS-treatment on macrophage activation, including activated Colanic Acid Building Blocks Biosynthesis, Arginine Biosynthesis and Citrulline-Nitric Oxide Cycle, LXR/RXR Activation, Acute Phase Response Signaling and Complement System in LPS-activated macrophages (Figure 1B, Supplementary Figure 3B). The levels of acylcarnitines were decreased in LPS-treated cells, suggesting that LPS treatment compromised fatty acid β-oxidation in macrophages (Figure 1C). This was also validated by the proteomic results, showing that carnitine O-palmitoyltransferase 1 (CPT1A) was down-regulated in LPS-treated macrophages (Supplementary Figure 3C). CPT1 catalyzes the rate limiting step in long-chain fatty acid oxidation (LCFA) by converting LCFA to acylcarnitines (Bonnefont et al., 2004). CPT1 has been implicated in regulating mitochondrial function and redox imbalance. Noticeably, the NADPH levels were increased by 5 folds in LPS-treated cell as compared with untreated cells in our metabolomics data (Figure 1E). Increasing NADPH was crucial for macrophage activation for generation of ROS and RNS, in which iNOS was upregulated in activated macrophages in proteomics results. It was expected that LPS treatment promoted the conversion of NAD(H) to NADP(H). Indeed, the cellular NAD^+^ level was decreased in LPS-treated cells, suggesting that less NAD^+^ was available for Sirtuins which used NAD^+^ to deacetylate targeted proteins and supplementation of NAD^+^ precursors can increase the NAD^+^ level and enhanced Sirtuin activities. This was confirmed by our results, showing that NMN replenishment increased the cellular NAD^+^ level and antagonized the expression of IL-6 and IL-1β in LPS-treated macrophages (Figure 2C, 3A–F).

PGE_2_ is known as a lipid mediator to exacerbates inflammation by inducing IL-6 production (Hinson et al., 1996; Kawahara et al., 2015). Our recent study revealed that NMN supplementation decrease PGE_2_ production via stabilizing 15-PGDH from oxidative degradation that alleviated liver fibrosis (Wang et al., 2018; Zong et al., 2021). However, NMN treatment in macrophages didn’t change the cellular 15-PGDH levels (data not shown). Rather, our data shows that COX-2 expression was decreased in LPS/NMN co-treated cells in comparison with LPS-treated cells, leading to decreased PGE_2_ production. We further demonstrated that NMN relieved LPS-induced inflammation via COX-2-PGE_2_ axis in COX-2 knockdown or overexpressing cells. The *Cox2* gene expression is regulated by gene transcription or post-transcriptional events. (Harper et al., 2008). Our data showed that NMN supplementation has no effect on COX-2 degradation. Furthermore, we demonstrated that other NAD precursors NAM and NR also downregulated COX-2 expression and PGE_2_ production, suggesting that the increase of NAD^+^ content was correlated with the decrease of COX-2 expression. The molecular mechanisms underlying NMN-mediated COX-2 downregulation is under further exploration.

In conclusion, we showed that altered NAD^+^ metabolism was a characteristic in activated macrophages and NMN supplementation suppressed pro-inflammatory cytokine production in LPS-induced inflammation. We revealed that the NMN supplementation decreased COX-2 expression and inhibited PGE_2_ production. These results suggest NMN replenishment is an effective approach for chronic inflammation treatment.

## Data Availability

The original contributions presented in the study are included in the article/Supplementary Material, further inquiries can be directed to the corresponding author. The data presented in the study are deposited in the (https://doi.org/10.3389/fmolb.2021.702107) repository.

## Abbreviations

NMN, Nicotinamide mononucleotide; NAD, Nicotinamide adenine dinucleotide; NR, Nicotinamide riboside; IL-6, Interleukin-6; TNF-α, Tumor necrosis factor-α; PGE_2_, Prostaglandin E_2_; COX-2, Cyclooxygenase-2; CPTIA, Carnitine O-palmitoyltransferase 1; TMT, Tandem mass tag; RT-qPCR, Real-time quantitative PCR; LPS, Lipopolysaccharide; NA, Nicotinic acid; NAM, Nicotinamide; IL-1β, Interleukin-1 beta; NO, Nitric oxide; COX-1, Cyclooxygenase-1; NSAIDs, Non-steroidal anti-inflammatory drugs; LCFA, Long-chain fatty acid oxidation; LC-MS, Liquid chromatography coupled to a mass spectrometry; ROS, Reactive oxygen species.
