# Supplementary material for: Nicotinamide Mononucleotide Alleviates LPS-Induced Inflammation and Oxidative Stress via Decreasing COX-2 Expression in Macrophages
Source: Front Mol Biosci. 2021 Jul 6;8:702107. doi: 10.3389/fmolb.2021.702107 (PMC8290259; doi:10.3389/fmolb.2021.702107)
Supplement: Supplementary file 1 [file DataSheet2.docx]

**Nicotinamide Mononucleotide Alleviates LPS-induced Inflammation and Oxidative Stress via Decreasing COX-2 Expression in Macrophages**

**Jing Liu^1†^, Zhaoyun Zong^1†^, Wenhao Zhang^1^, Yuling Chen^1^, Xueying Wang^1^,** **Jie Shen^2^, Changmei Yang^1^, Xiaohui Liu^1^, Haiteng Deng^1*^**

**^1^** MOE Key Laboratory of Bioinformatics, Center for Synthetic and Systematic Biology, School of Life Sciences, Tsinghua University, Beijing, China

^2^ Shenzhen Hope Life Biotechnology Co., LTD, Shenzhen, China

^†^ These authors contributed equally to this work.

* **Correspondence：**

Corresponding Author

[dht@mail.tsinghua.edu.cn](mailto:dht@mail.tsinghua.edu.cn)


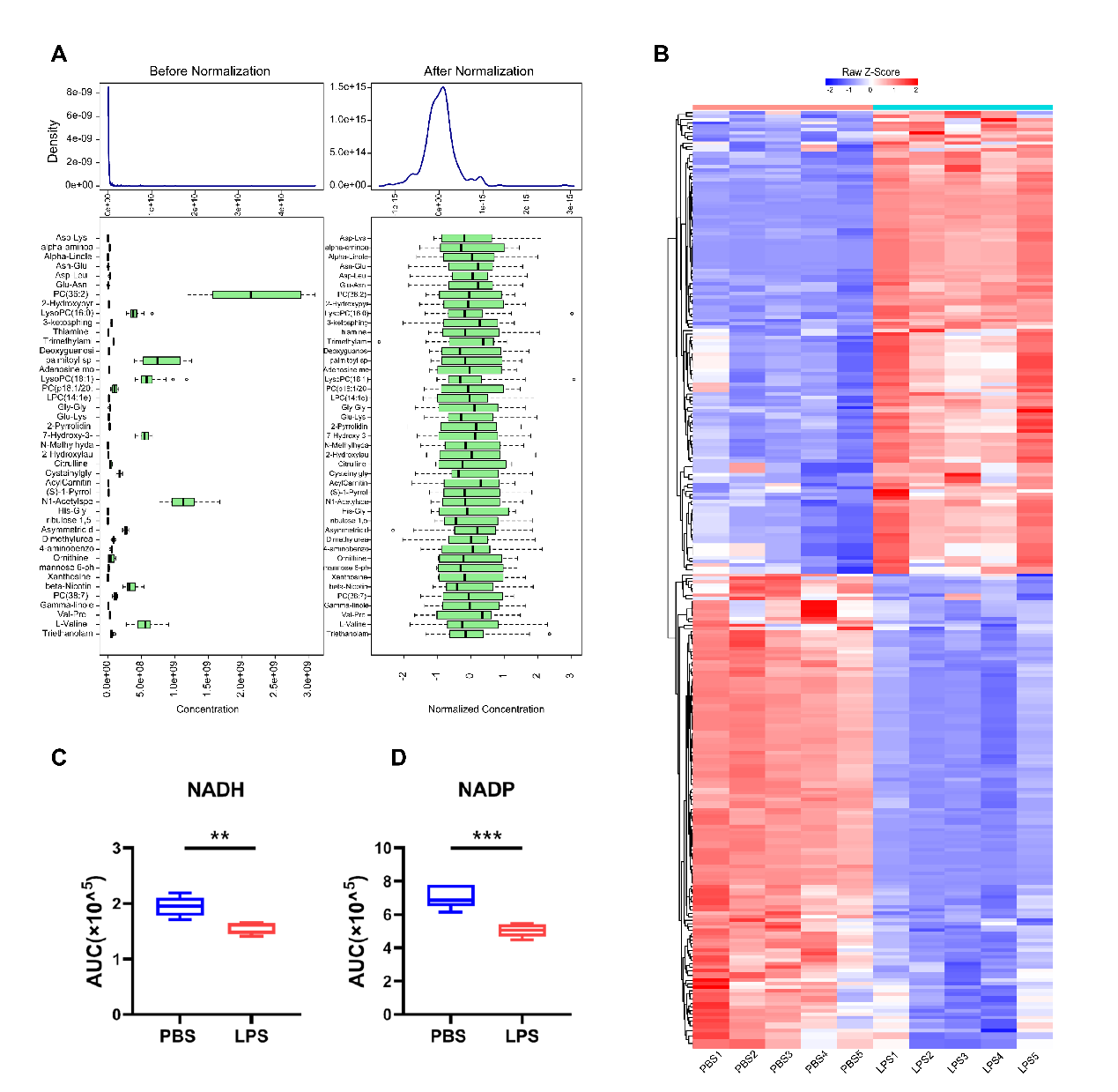


**Figure S1.** Data processing and Heatmap analysis of all metabolites in LPS-treated RAW264.7 cells as compared with those in untreated cells.

(A) The boxplots and the density plots of 50 features were performed before and after normalization by Z-score calculation.

(B) Heatmap analysis of metabolites in LPS-treated cells as compared with control group after normalization. The results show that the different patterns of the metabolites in metabolomics analysis.

(C and D) NADH and NADP levels from the metabolomics data (**** p < 0.0001, ** p < 0.01, * p < 0.05, n = 5, mean ± SEM).


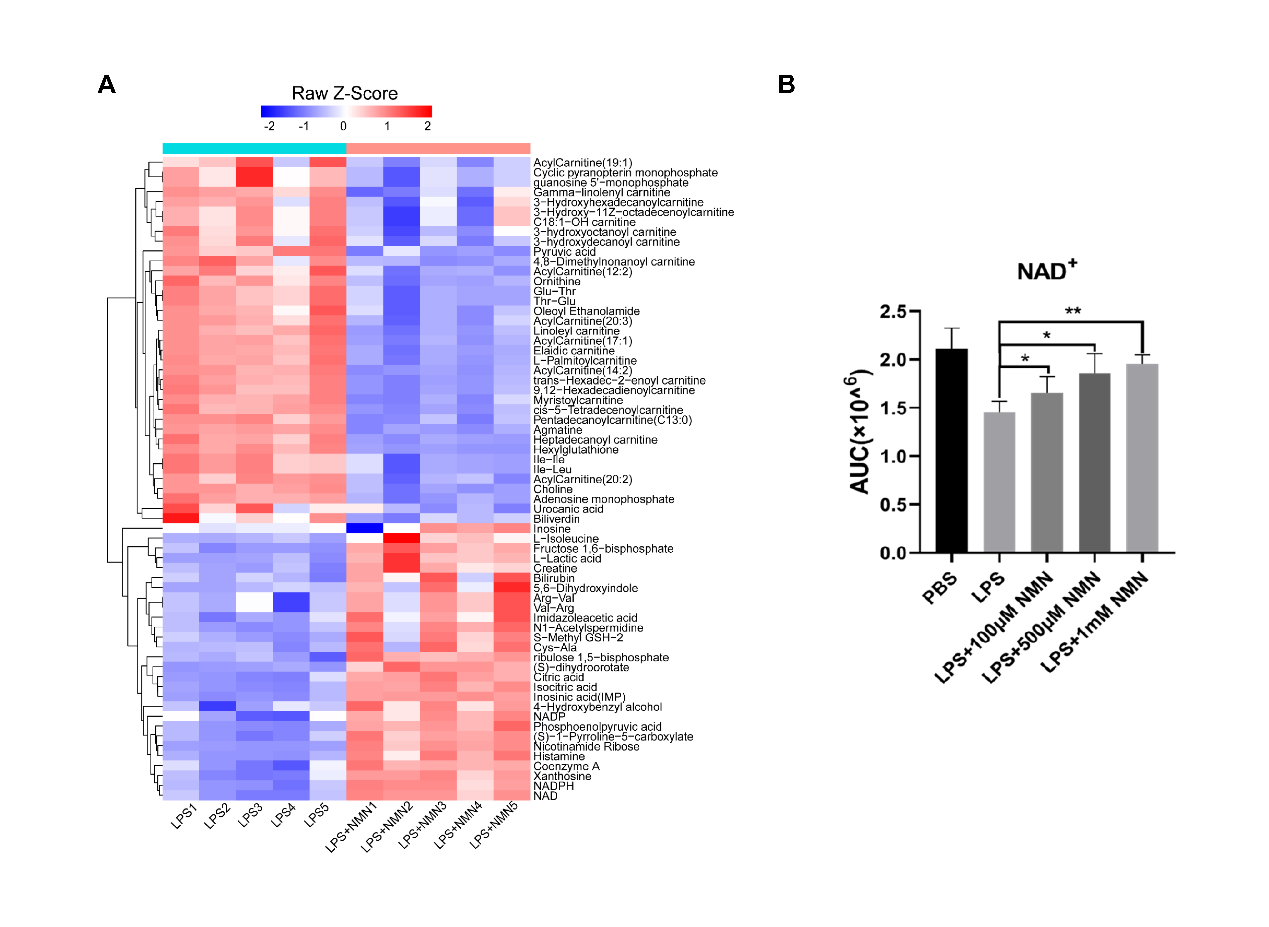


**Figure S2.** NMN treatment induced alternations in metabolites as compared with LPS-induced RAW264.7 cells.

(A) Heatmap analysis of metabolites in LPS+NMN treated cells as compared with LPS-treated group after normalization. The results show that the different patterns of the metabolites in metabolomics analysis.

(B) The contents of NAD+ in untreated, LPS (100 ng/mL)-treated and LPS-NMN (100μM/500µM/1mM)

co-treated RAW264.7 cells were analyzed by LC-MS/MS (n=4). P values were calculated using one-way ANOVA test; **** p < 0.0001, ** p < 0.01, * p < 0.05; Data are shown as means ± SEM.


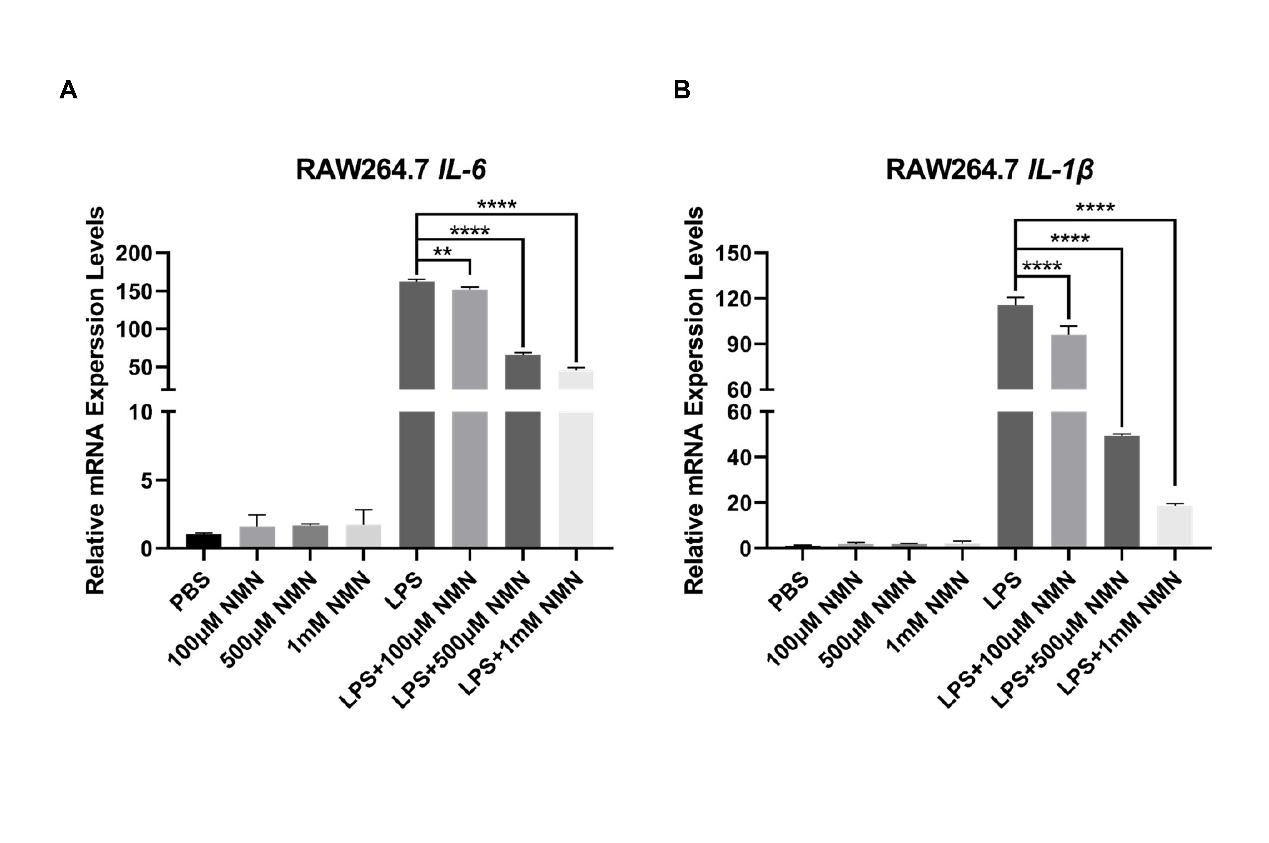


**Figure S3.** Effect of different concentrations of NMN on the mRNA expression of *IL-6* and *IL-1β* in RAW264.7 cells

(A) Relative mRNA expression of *IL-6*.

(B) Relative mRNA expression of *IL-1β*.

P values were calculated using one-way ANOVA test. **** p < 0.0001, ** p < 0.01, * p < 0.05; mean ± SEM.


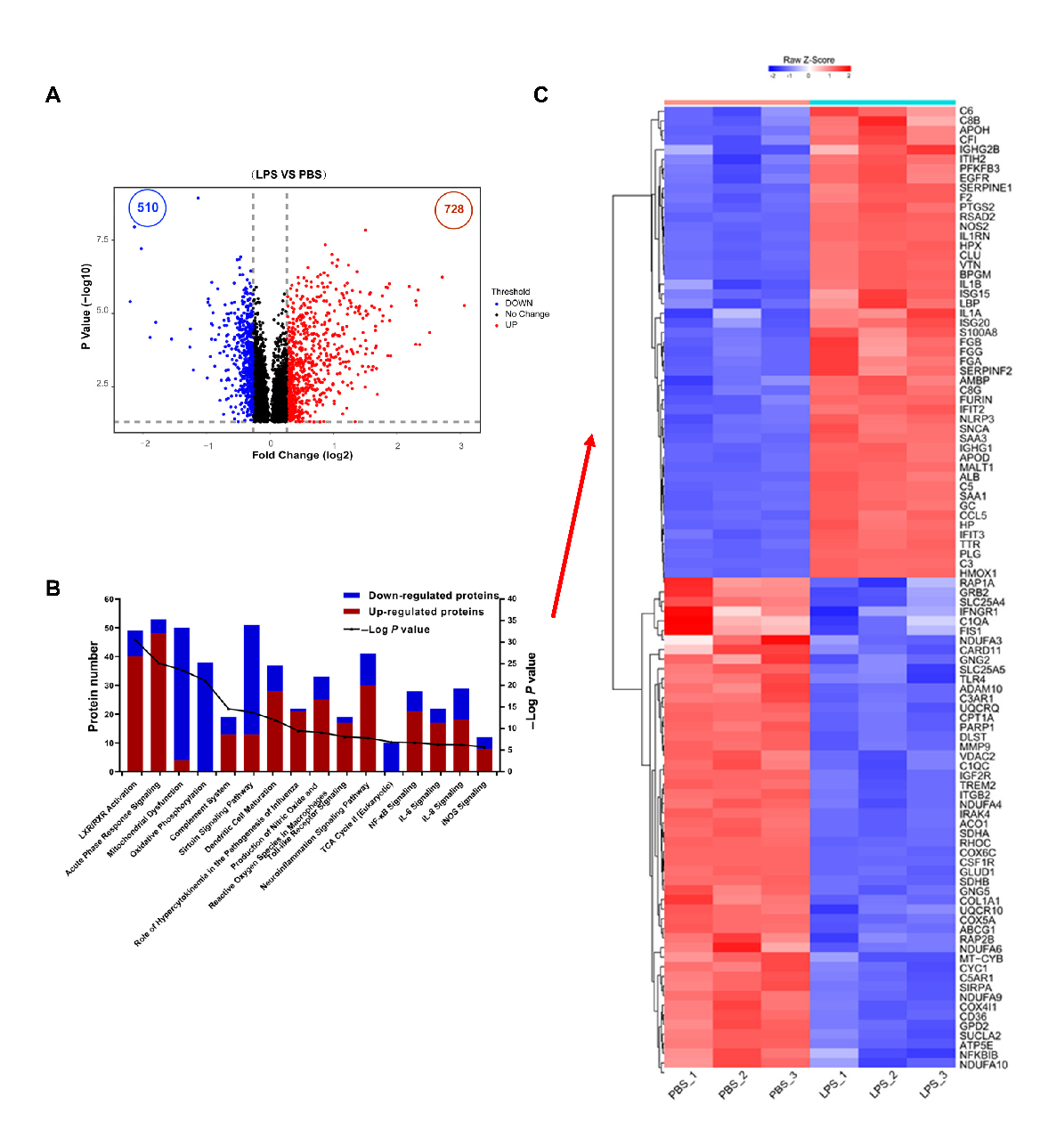


**Figure S4.** LPS-mediated proteome changes in RAW264.7 cells.

(A) The volcano plot shows differentially expressed proteins (DEPs) in LPS-treated cells as compared with untreated cells. The red and blue dots indicate the significantly changed proteins with fold-change > 1.2 or < 0.83 and p value < 0.05.

(B) Functional enrichment analysis using the differentially expressed proteins with IPA. The red bar represented up-regulated proteins, and blue represented down-regulated; (LPS-treated cells versus untreated cells> 1.2-fold or< 0.83, P < 0.05).

(C) Heatmap clustering analysis of the differential expressed proteins selected based on IPA analysis in LPS-treated cell compared with untreated.


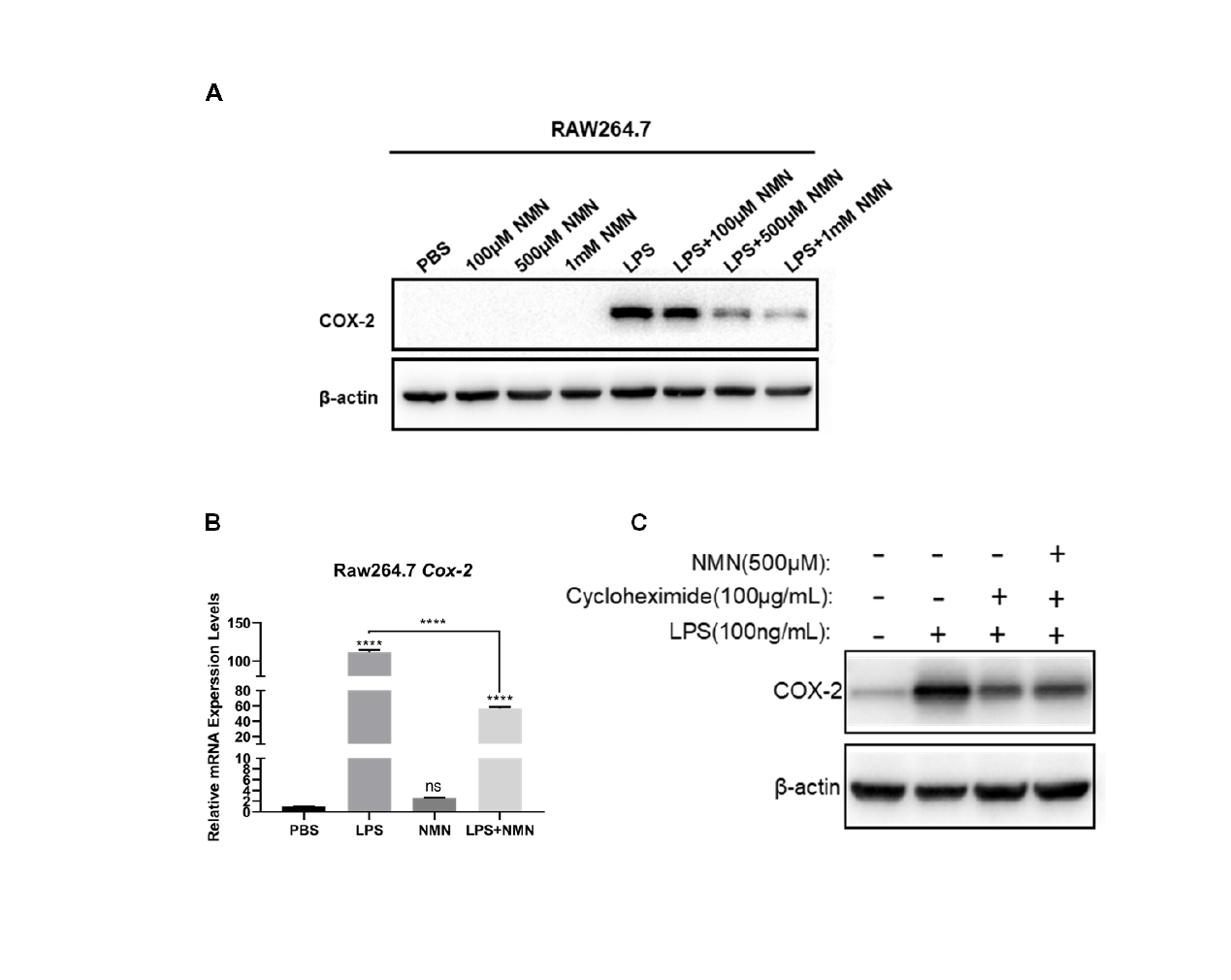


**Figure S5.** NMN decreased cellular COX-2 expression in macrophages.

(A) COX-2 expression in RAW264.7 cells was analyzed by western blotting.

(B) The mRNA expression of COX-2 decreased in LPS-NMN-treated RAW264.7 cells as compared with LPS-treated cells, detected by RT-qPCR(n=3). P values were calculated using one-way ANOVA test; **** p < 0.0001, ** p < 0.01, * p < 0.05; Data are shown as means ± SEM.

(C) COX-2 levels were analyzed by western blotting. RAW264.7 cells were treated with NMN (500µM), LPS (100ng/ml) and cycloheximide (100μg/mL) (**** p < 0.0001, ** p < 0.01, * p < 0.05, n = 3, mean ± SEM).


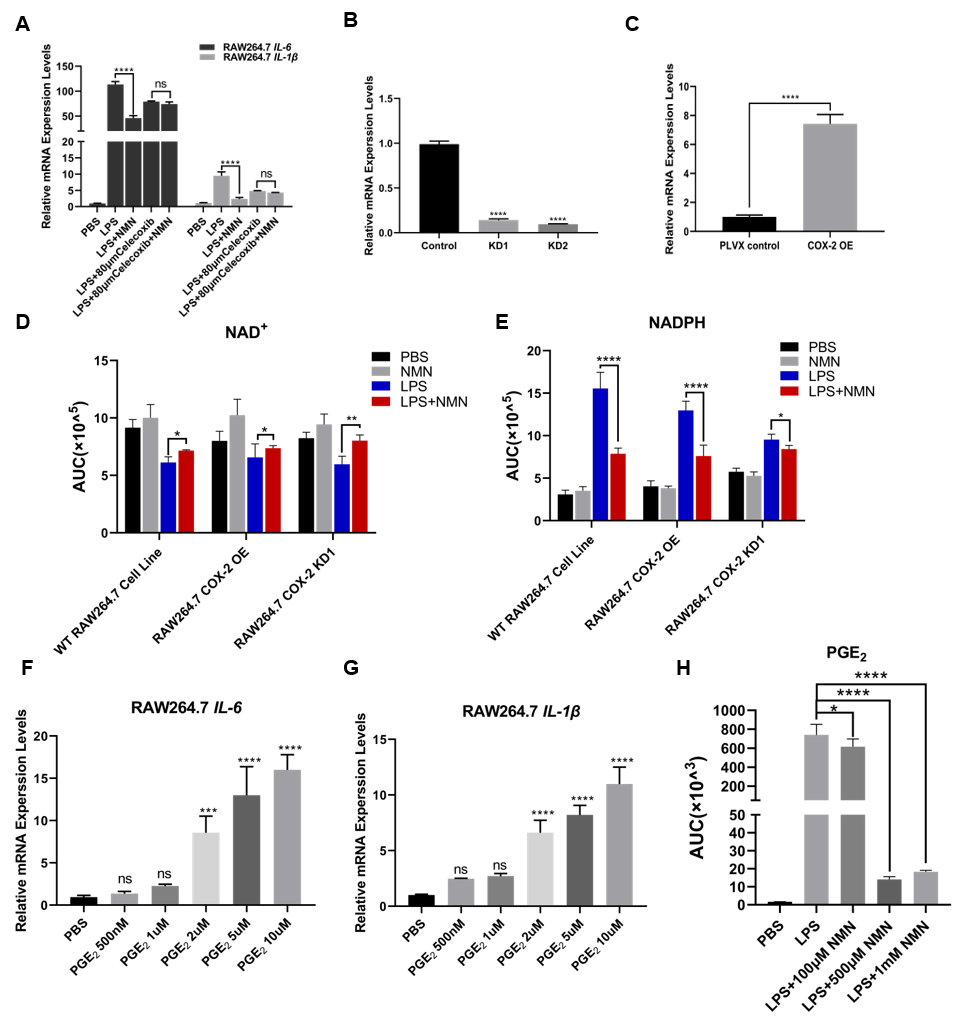


**Figure S6.**NMN alleviated LPS-induced Inflammation via decreasing COX-2 expression to inhibit prostaglandin E2 synthesis.

(A)The mRNA expression levels of IL-6 and IL-1β in RAW264.7 cells were detected by RT-qPCR(n=3). The RAW264.7 cells were treated with LPS, NMN and celecoxib, respectively.

(B-C) The mRNA expression of *Cox-2* in RAW264.7-COX-2-KD cell lines or RAW264.7- COX-2-OE cell line was detected by RT-qPCR compared with control cells(n=3).

(D-E) The contents of NAD^+^ and NADPH in COX-2-OE-cells, COX-2-KD-cells and wild-type RAW264.7 cells were analyzed by LC-MS/MS (n=4). P values were calculated using one-way ANOVA test; **** p < 0.0001, ** p < 0.01, * p < 0.05; Data are shown as means ± SEM.

(F-G) The mRNA expression of *IL-6* and *IL-1β* in RAW264.7 cell at different concentrations of PGE_2_ are shown (n=3). P values were calculated using one-way ANOVA test; **** p < 0.0001, ** p < 0.01, * p < 0.05; Data are shown as means ± SEM.

(H) The levels of PGE2 in RAW264.7 cells were analyzed by LC-MS/MS (n=4).

P values were calculated using one-way ANOVA test; **** p < 0.0001, ** p < 0.01, * p < 0.05; Data are shown as means ± SEM.
